# Supplementary material for: Kinetics and seroprevalence of SARS-CoV-2 antibodies: a comparison of 3 different assays
Source: Sci Rep. 2021 Jul 21;11:14893. doi: 10.1038/s41598-021-94453-5 (PMC8295301; doi:10.1038/s41598-021-94453-5)
Supplement: Supplementary file 1 — Supplementary Information. [file 41598_2021_94453_MOESM1_ESM.docx]

**Supplemental Table 1: Specification of travel history and symptoms**

|  | **Percentage** | **Destination** |
| --- | --- | --- |
| **travel history** | 21  26  <5 | Austria  Italy  United States, Turkey, Australia, South Africa, France |
|  |  |  |
|  | **Percentage** | **Symptoms** |
| **Symptoms** | 80  64  60  54  47  41  32  13 | cough  headache  fever  muscle/limb pain  rhinitis  dyspnea  sore throat  nausea/vomiting |

Percentages for destination based on all participants who reported a travel history, percentages for symptoms based on all participants who reported symptoms.
